# A Review of Brain-Inspired Cognition and Navigation Technology for Mobile Robots

**DOI:** 10.34133/cbsystems.0128

**Published:** 2024-06-27

**Authors:** Yanan Bai, Shiliang Shao, Jin Zhang, Xianzhe Zhao, Chuxi Fang, Ting Wang, Yongliang Wang, Hai Zhao

**Affiliations:** ^1^ School of Computer Science and Engineering, Northeastern University, Shenyang 110819, China.; ^2^ State Key Laboratory of Robotics, Shenyang Institute of Automation, Chinese Academy of Sciences, Shenyang 110016, China.; ^3^ Institutes for Robotics and Intelligent Manufacturing, Chinese Academy of Sciences, Shenyang 110169, China.; ^4^ Department of Artificial Intelligence, University of Groningen, Groningen 9747 AG, Netherlands.

## Abstract

Brain-inspired navigation technologies combine environmental perception, spatial cognition, and target navigation to create a comprehensive navigation research system. Researchers have used various sensors to gather environmental data and enhance environmental perception using multimodal information fusion. In spatial cognition, a neural network model is used to simulate the navigation mechanism of the animal brain and to construct an environmental cognition map. However, existing models face challenges in achieving high navigation success rate and efficiency. In addition, the limited incorporation of navigation mechanisms borrowed from animal brains necessitates further exploration. On the basis of the brain-inspired navigation process, this paper launched a systematic study on brain-inspired environment perception, brain-inspired spatial cognition, and goal-based navigation in brain-inspired navigation, which provides a new classification of brain-inspired cognition and navigation techniques and a theoretical basis for subsequent experimental studies. In the future, brain-inspired navigation technology should learn from more perfect brain-inspired mechanisms to improve its generalization ability and be simultaneously applied to large-scale distributed intelligent body cluster navigation. The multidisciplinary nature of brain-inspired navigation technology presents challenges, and multidisciplinary scholars must cooperate to promote the development of this technology.

## Introduction

**Fig. 1.**
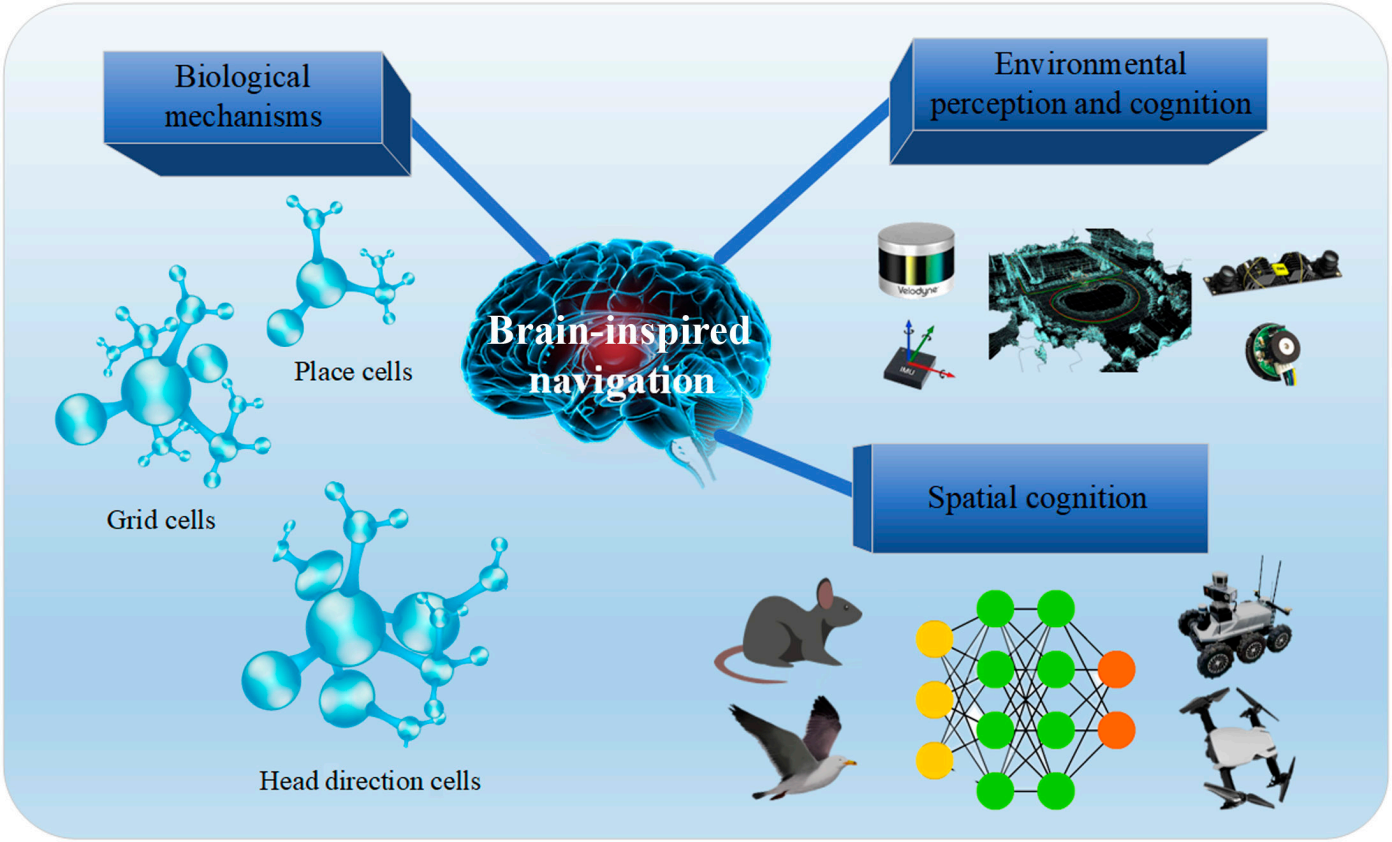
Overview of the brain-inspired technology for mobile robots.

Numerous animals in nature have been known to have excellent navigational abilities to achieve self-localization in complex and unknown environments and to accomplish basic tasks through empirical navigation. Currently, the autonomous navigation of robots is a major area of interest in the research field. Recent advances in neuroscience have unveiled the mechanisms underlying animal brain navigation. On the basis of the neural structure of animal brains and their information-processing mechanisms, researchers have proposed a novel biomimetic intelligent navigation algorithm for unknown and complex environments, called brain-inspired navigation. An overview of brain-inspired technology for mobile robots is shown in Fig. 1.

Brain-inspired navigation is a bionic intelligent navigation technique that draws inspiration from the spatial cognitive neural mechanisms of the animal brain and incorporates the neural computational mechanisms of learning and memory into traditional robot navigation techniques. This technology improves the robustness, autonomy, and environmental self-adaptation of the robotic navigation systems. It can characterize the spatial orientation of an intelligent body based on the spatial navigation mechanism of the brain, learn a cognitive map of the environment, and cope with path planning and task decision-making in unstructured environments.

Brain-inspired navigation technology establishes a multilayered modular network model of perception and memory and utilizes a series of mechanisms, such as the brain’s integration of multisensory information, neural dynamics, neural coding, and synaptic plasticity, to constrain the structure and function of the model, such that it can integrate perceptual and motor information and form a cognitive map representation. The advantages of this technique include the cognitive ability of spatial relations, efficiency and robustness of localization, adaptability to environmental changes, and the scalability of map representations.

Compared with traditional robot navigation, brain-inspired navigation has the advantages of low energy consumption, high robustness, and strong resilience in unknown and complex environments. The major contributions of this study are as follows.

1. This study describes the biological mechanisms involved in brain-inspired navigation, particularly the different divisions of labor among different navigational cells in the hippocampus of animal brains during a single navigation task. It summarizes the biological mechanisms of 3 key navigational cells currently being studied in brain-inspired navigation techniques: the place cell (PC), grid cell (GC), and head direction cell (HDC). These cells play a crucial role in navigation and provide an important biological basis for the development of brain-inspired navigation technologies.
2. In this study, brain-inspired navigation technology for unknown complex environments is introduced from the perspective of the processes experienced in navigation tasks, including the 3 processes of brain-inspired environment perception, brain-inspired spatial cognition, and target-oriented navigation. On the basis of the brain-inspired biological mechanism and realization navigation process, this paper summarizes the realization of 3 processes: environmental perception, spatial cognition, and goal-oriented navigation. In addition, it summarizes the research status and achievements of related technologies from different perspectives, analyzes the advantages and limitations of these technologies, and explains their brain-inspired biological mechanisms referenced by them. At the end of the paper, according to the different navigation cells realized in the model used by the technology, the related research is summarized. These results provide important theoretical support and practical guidance for the development of brain-inspired navigation technologies.
3. This study analyzes state-of-the-art brain-inspired navigation technologies in unknown, complex environments. It discusses the developmental trends in environmental perception, spatial cognition, and target navigation technology for unknown complex environments that could be discussed in the future.

## Biological Mechanisms

**Fig. 2.**
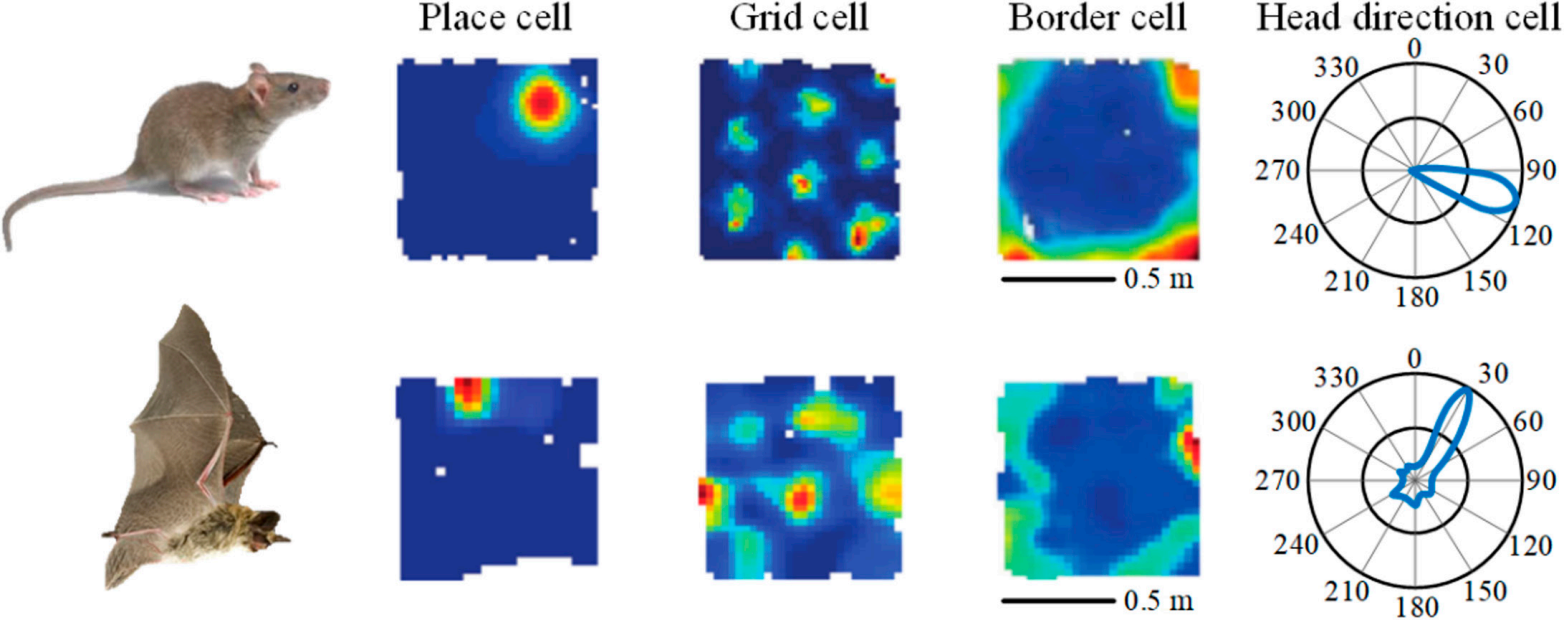
Biological mechanisms of brain-inspired navigation.

With continuous developments in brain science and cognitive neuroscience, researchers have gradually revealed the navigational mechanisms of the insect and mammalian brains [1]. The navigational activity of mammals is closely related to neurons in the hippocampus and rhinal cortex of the brain. The neurons associated with navigation in the mammalian hippocampus include PCs, HDCs, GCs, velocity cells (VCs), boundary cells (BCs), and action cells (ACs), as shown in Fig. 2. The navigation completion process is based on the firing activity of a large number of navigation cells in clusters that form neural circuits for navigation and positioning calculations. Through the division of labor, these cells enable animals to achieve efficient spatial cognition and precise matching navigation of scene targets in unknown and complex environments. In the navigation task, specific HDC discharge activation characterizes orientation information, PCs encode spatial location, GCs perform path integration and construct cognitive maps, VCs control speed, BCs encode the distance and direction of obstacles, and ACs enable the animal to generate decision responses. In the navigation task, the organism first senses its movement information according to the information sensed by the sensory organs on the body, activates the head discharge toward the cell, estimates its own orientation and forward speed, and transmits it to the GCs. GCs integrate the information, establish the discharge characteristics of hexagonal structures in the space environment, and build a spatial scale map; PCs determine the location according to all the received environmental information combined with coding. In the presence of a navigational target, dopamine neurons are activated, secreting dopamine to create a reward for ACs to output action instructions, and finally control the body to move toward the target. Thus, PC, GC, and HDC are the 3 basic cell types in the hippocampus of the animal brain and are an important basis for brain-inspired navigation research.

### Place cells

In 1971, O’Keefe et al [2] discovered that the firing frequency of certain nerve cells increases when they move to a specific position, resulting in the emergence of positional cells. Subsequently, PCs have been found in the brains of monkeys [3] and humans [4]. PCs are useful for helping animals that spatially localize themselves when completing navigation tasks and encoding a specific position of the animal in the environment. Firing activity strongly correlated with the spatial location of the animal. Under dark conditions or when reentering a given position after several months, the position cell discharge pattern remains essentially the same as the original [5]. When the external environmental space is slightly changed, the discharge position of the PCs remains unchanged, and only the discharge frequency in the position field is changed for characterization [6]. If a new environment is entered, the center of the positional field of the cell changes to characterize the new position node [7]. The stable encoding of animal spatial locations by PCs is the neural basis for mammalian spatial cognitive navigation.

### Grid cells

Following the discovery of positional cells, researchers identified GCs in the medial entorhinal cortex of rats. Similarly, subsequent studies have found GCs in the brains of monkeys [8] and humans [9]. GCs assist animals in constructing cognitive maps. In the active regions, PCs must correspond to a specific region to generate discharges, whereas GCs can generate discharges in multiple regions. In unknown environments, GCs rapidly form discharge fields and maintain their stability, while integrating movement paths with exogenous and self-motion information as inputs. GCs take advantage of the multiscale property to utilize large-scale navigation over a large range and then utilize small-scale guidance near the destination [10], considering the minimum spatial uncertainty and highest accuracy to achieve low-energy, high-precision, and robust navigation for animals [11].

### Head direction cells

In 1984, Taube et al. [12] identified HDCs in the rat hippocampus that were selectively discharged in response to head orientation. HDCs are present in the mammalian brain [13]. HDCs function as compasses, encoding information about an animal’s orientation in space by integrating endogenous and exogenous information, each of which encodes only a specific inversion. The cells discharge more strongly when the angle between the animal’s head orientation and the direction in the HDC population is smaller [14]. HDCs encode orientation mainly from the information input of the vestibular system. In the absence of external stimuli, they still encode the current orientation using self-motor information. Changes in the head orientation lead to corresponding rotations in the discharge field of the PCs; however, the encoding of the PCs does not require an intact HDC population. The collaboration of head-oriented cells with other navigation cells, such as PCs and GCs, enables animals to navigate in unknown and complex environments.

## Brain-Inspired Environmental Perception

**Fig. 3.**
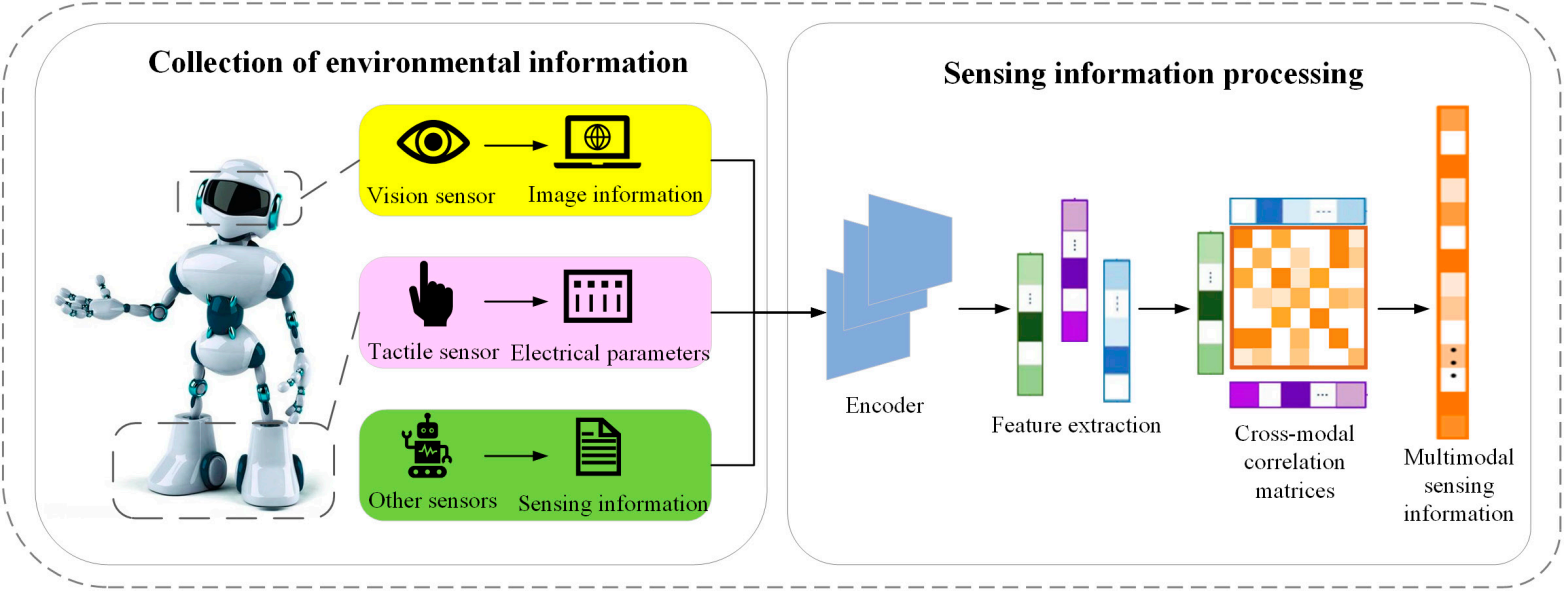
Brain-inspired environmental perception model.

Animals rely on various sensory organs and sensory-information-processing mechanisms to perceive their surrounding environment. Brain-inspired environment perception realizes multidimensional feature extraction of the carrier’s navigation information and the surrounding environment by drawing on the rich biological structure of the animal’s visual, tactile, olfactory, and auditory sensory organs and its multimodal-sensory-information-processing mechanism. Figure 3 shows the brain-inspired environmental perception process, which includes 2 parts: environmental information collection and sensing information processing. Environmental perception information includes visual, tactile, and other sensor information, such as robot odometer data. In information processing, various encoders are first used for encoding, and then a multimodal correlation matrix analysis is performed on the obtained features to obtain the multimodal sensing information.

### Environmental information collection

Prior to the navigation task, various modal bionic sensors were used to collect information regarding the surrounding environment. Bionic navigation sensors include optical, mechanical, and multimodal-fusion-based devices. These navigation sensors draw on the mechanism of animal organs sensing the natural environment to form navigation information and the mechanism of navigational cells in the brain to process the information. This can transform the information sources of the natural world, such as light, magnetism, and scene characteristics, into navigation information of the carrier’s movement, such as heading, position, speed, and attitude, and have the characteristics of full autonomy, anti-interference, and measurement errors that do not accumulate with time. It is characterized by full autonomy and anti-interference, and measurement errors do not accumulate over time.

The most commonly used sensor mode for autonomous navigation is vision; depth perception using a red–green–blue-depth (RGB-D) camera or a light detection and ranging (LiDAR) sensor is also commonly used. Animal visual sensory mechanisms have been developed into optical-based navigation sensors. Insect compound eyes are arranged in curved arrays with a sub-eye field of view angles capable of reaching the full field of view, giving the compound eye a unique advantage in environmental perception. Park et al. [15] proposed an integrated low-power 2D (2-dimensional) optical flow sensor that can be configured in a semihemispherical platform, realizing an artificial compound eye that can provide wide-field-of-view sensing capability for the autonomous navigation of microvehicles. During navigation, an increasing number of computer vision tasks, such as object tracking and obstacle avoidance, must be performed. To realize such functions, it is crucial to extract temporal and spatial information from a scene. The traditional imaging system consists of sensors and processors and contains excessive redundant data when extracting information from a scene, resulting in high system power consumption, which is inadvisable on edge devices. The multimode vision sensor proposed by Chiu et al. [16] consists of an imaging sensor and a back-end image signal processor. Through processing-in-sensor technology, computing power is directly integrated into the sensor. The system can selectively transmit basic features without transmitting raw data, thereby minimizing redundant data and achieving high energy efficiency and low latency.

In addition to visual estimation, humans and animals use the tactile sensations of their skin and muscles to sense their surroundings. In scenes with poor lighting conditions, the effectiveness of visual sensors is limited, and other types of sensors must be used to realize environmental perception. This animal tactile-sensing mechanism provides a reference for developing mechanical sensors. Bionic tactile sensors, which are based on the mechanism of animal tactile perception of object materials and spatial properties, can perceive object space and objects, compensate for the lack of visual sensing, and realize a fine perception of the environment. Shukla et al. [17] proposed a small tactile sensor inspired by the whiskers in rodent models. Its 360° tactile view and force-sensing capabilities make it suitable for navigation in limited and narrow spaces. It can help mobile robots made of Semmes–Weinstein monofilaments to navigate in vacuum, narrow, or blind areas. Simultaneously, the tactile sensor may also help detect the texture and flexibility of obstacles. Among some legged robots that also use a sense of touch when collecting sensing information, DogTouch, a new 4-legged robot with tactile sensing feet (TSF) proposed by Weerakkodi Mudalige et al. [18], can move in unknown environments by recognizing specific tactile patterns. To address the problem of vehicles’ inability to navigate visually in complex unknown environments, such as darkness or smoke, Borkar et al. [19] proposed an autonomous navigation method that relies on haptic feedback. This method uses an array of force/contact sensors on a quadrotor to determine local obstacle geometries, track the contours of the sensed objects, and navigate through complex, unknown environments.

In addition, the fusion of different modal sensors can perceive the environment at different scale layers and provide rich self-motion information. Weerakoon et al. [20] utilized different modal observations to train their predictive model, including RGB cameras, 3D LiDAR, and robotic odometers, to project high-dimensional sensor inputs into feature vectors and express them as feature maps to train their model. By using multisensor inputs for navigation through sensor fusion, potential collisions and malfunctions can be avoided.

In environmental perception, the most important sensor mode is vision, which directly obtains environmental sensing information, such as images. LiDAR and robot odometer sensors have also been used to measure environmental perceptions. However, in environments where the visibility is poor because of dust, smoke, or darkness, vision-based navigation is not possible, and haptic navigation is achieved using force/contact sensors. Using the sense of touch, road surface textures can be identified, and information about obstacles can be determined to achieve safe and intelligent navigation. Moreover, the tactile sensor is lighter than the LiDAR, which is more suitable for the navigation of aircraft and drones. The combination of visual, tactile, and other multimodal sensors can improve environmental perception. For environmental perception in the autonomous navigation of unknown environments, more sensor modes can be integrated, including vision, touch, hearing, and temperature, which can be added to special environments to achieve more control functions.

### Sensor information processing

In the face of massive amounts of sensing information, learning from animal-sensing information-processing mechanisms to quickly and accurately realize scene perception, target recognition, and feature extraction is another important problem in brain-inspired environment perception. In recent years, machine learning technologies, such as support vector machines [21] and convolutional neural networks (CNNs) [22], have been used in navigation to identify and locate surrounding objects. Simultaneously, the processing of visual-sensing information can be improved by integrating various technologies. Sun et al. [23] applied a feature-matching algorithm composed of multilevel matching modules comprising *k*-nearest neighbors, threshold filtering, a feature vector paradigm, and random sample consistency to vision-based navigation, thereby solving the problems of low efficiency, low robustness, and low feature matching accuracy. In addition, it improves the processing efficiency of visual sensing information. Deep neural networks (DNN) transform high-dimensional complex sensory data into concise representations of visual features, thereby improving the recognition and classification performance. Neural coding is indispensable in the brain’s information-processing systems. The neural coding framework is established using a DNN, and the visual stimulus is encoded into hierarchical intermediate features [24] using a DNN so that the agent can better use the image information to determine its position. In addition, Plebe et al. [25], inspired by how human cognition and vision perceive the environment, proposed occupancy grid mapping, which is an abstract representation of the driving environment in autonomous driving. They added cognitive rationality to the occupancy grid mapping task to mimic how human perceptual systems process information; however, this method lacks dynamic information. It does not integrate information regarding the movement of objects into the model, which differs from the human cognitive mechanisms.

When processing multimodal sensing information, different types of modal information must be processed separately and then combined for analysis, which is more difficult than processing a single type of sensing information. Fazeli et al. [26] proposed a multisensory fusion complex operation model based on hierarchical learning, which can capture descriptive underlying structures. The robot learns the relevant probability models of touch and vision through a short exploration phase to realize multimodal sensing and information processing. The MM-NeuroPos neural-inspired positioning system proposed by Liu et al. [27] integrates microelectromechanical systems (MEMS) sensors and digital terrestrial multimedia broadcast signals and realizes collaborative positioning by integrating a variety of cells of different scales, which can be used as a complementary solution to global navigation satellite systems.

To process sensing information, efficient utilization of environmental perception information is crucial. To reduce data redundancy, a certain amount of computing power can be integrated into a sensor that collects environmental perception information, and certain feature extractions can be performed on the sensor. Different algorithms can be used to achieve further extraction, and environmental information can be simplified by imitating how the brain processes it. Simultaneously, imitating the mechanism of humans feeling the environment—the combination of touch, vision, and intuition to form a multimodal-information-processing mechanism—is key to sensor information processing. Currently, many processing algorithms lack such dynamics. Future developments could integrate dynamic information into the model to better support sensor information processing.

## Brain-Inspired Spatial Cognition

**Fig. 4.**
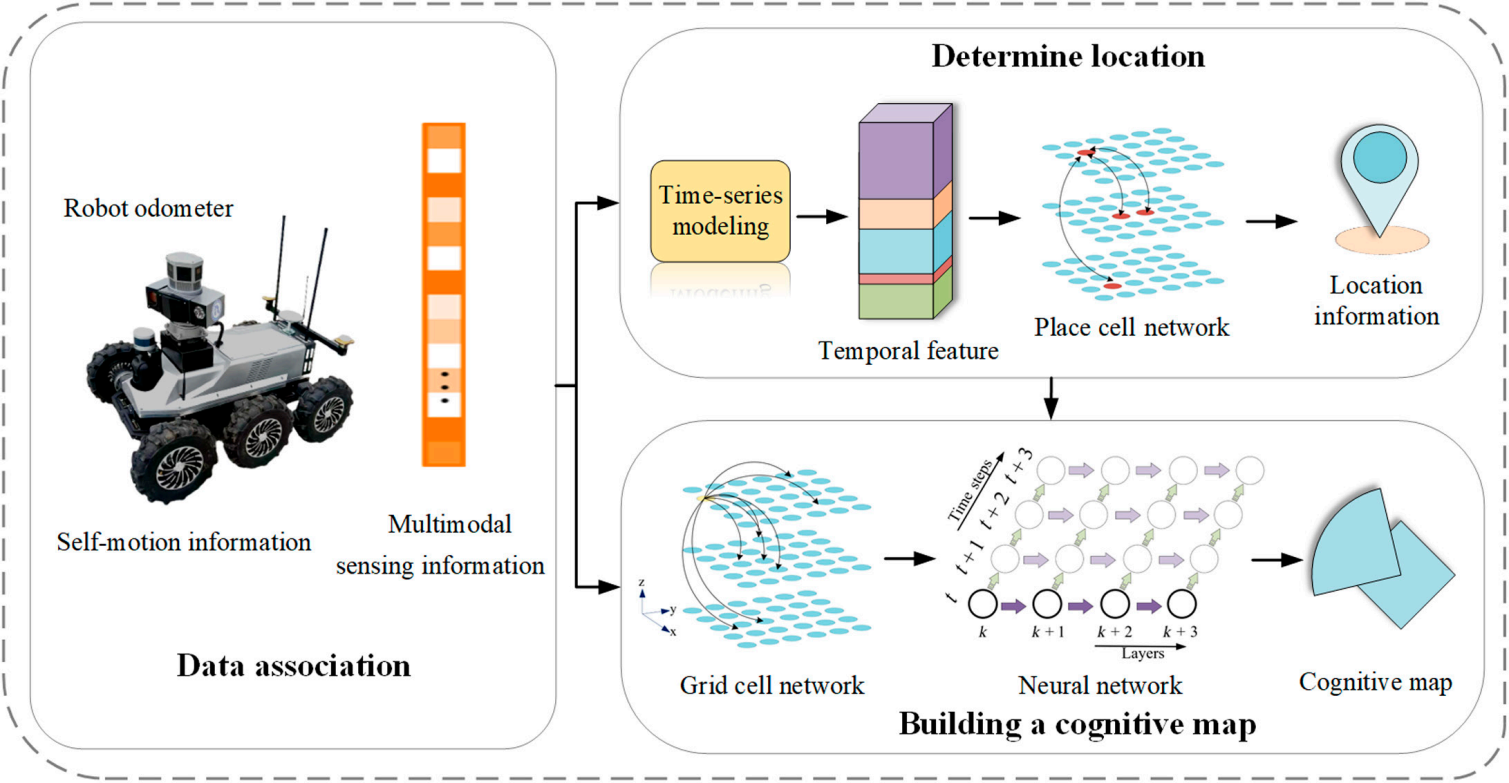
Brain-inspired spatial cognition model.

Cognition refers to the process of acquiring or applying knowledge, that is, information processing. The human brain receives information from the outside world, processes it, and transforms it into internal psychological activities that, in turn, govern human behavior. This process is known as information processing, also known as the cognitive process. Spatial awareness refers to the human ability to perceive, understand, and organize a spatial environment, which enables us to navigate through unknown environments and establish mental maps. Brain-inspired spatial cognition draws on the self-organized cluster firing mechanism of brain navigation cells to realize the navigation computation of location and environment information extracted from the brain-inspired environment perception process, including associated memory, storage of related information, and construction of cognitive maps. A brain-inspired spatial cognition model is illustrated in Fig. 4. Associate multimodal sensing information related to environmental information with self-motion information recorded by robot odometers use the correlated information to model time series to obtain spatiotemporal features, obtain self-position results through a PC network, combine position information, and construct a cognitive map through GC and neural networks. Neural computation of self-organized cluster discharge activities of navigation cells is the basis of brain-inspired spatial cognition. Deep learning (DL), continuous attractor neural network (CANN), and spiking neural network (SNN) models have been developed based on this mechanism.

### DL model

DL is a type of machine learning that discovers distributed feature representations of data by combining low-level features to form more abstract high-level layers that represent attribute categories or features. The motivation for studying DL is to build a neural network that simulates the human brain for analytical learning and the mechanisms by which the human brain interprets data. In recent years, DL functions have rapidly developed and are widely used in driving assistance, artificial intelligence, and autonomous robots. DL techniques are widely used to detect, recognize, and classify images. DL networks can be used to achieve spatial cognition in navigation tasks. Typical DL networks include radial basis function neural networks (RBFNNs), CNNs, long short-term memory (LSTM) neural networks, and generative adversarial networks (GANs). The RBFNN is a feedforward neural network with a unique best approximation (overcoming the local minimum problem), simple training, and fast learning convergence. It has been proven that RBFNN can approximate any continuous nonlinear network with arbitrary accuracy and is widely used in function approximation, speech recognition, pattern recognition, image processing, automatic control, and fault diagnosis. Li et al. [28] proposed a location representation method based on a rat hippocampal positioning mechanism. This method uses an RBFNN to associate landmarks extracted by the system with the location cognitive map and stimulates the discharge of location cells by sensing the distance information between these cells and different landmarks to achieve autonomous positioning. The basic module of a CNN is composed of input and output layers as well as multiple hidden layers, which are often used to analyze visual images. Compared with other image classification algorithms, CNNs use relatively little preprocessing. In semistructured and unstructured road navigation tasks, Li et al. [29] used deep CNNs for pixel-level segmentation to identify road areas and proposed a region-detection algorithm to ensure the movement of the robot in traversable areas. However, this algorithm is often used for visual navigation and lacks integrated information from other types of sensors. LSTM is a type of time-cyclic neural network that can solve the long-term dependence problems that exist in general recurrent neural networks. To make full use of time information in navigation tasks, Walch et al. [30] proposed a CNN with an LSTM neural network architecture, which is used for camera attitude regression through image context modeling and is helpful in improving the positioning performance to some extent. CNNs have been successfully applied in the fields of target recognition, image classification, and visual position recognition and can return images to fixed scenes. The ideal relative camera pose regressor can not only train and test in fixed scenes but also span different visible scenes in parallel. Yang et al. [31] proposed a cross-scene relative camera pose estimation method, RCPNet, based on a deep CNN to achieve positioning. This method successfully estimates the relative camera poses in various urban scenes using a single training model. A GAN comprises generative and discriminant models. The generative model captures the distribution of the sample data, and the discriminant model determines whether the input is real data or generated samples. GANs are widely used for image processing. In outdoor autonomous navigation, Sindhu and Saravanan [32] used a partial CNN and double interactive Wasserstein GAN to solve landmark detection and location problems, improve positioning accuracy, and reduce computing time.

Methods developed using DL can enhance spatial cognitive navigation. For example, a continuous graph attention network was used to realize an image-matching framework for continuous learning and solve the keypoint-matching problem in a 3D scene reconstruction system [33]. Keypoint matching is necessary to realize a scene model that is close to reality with different views. Taking advantage of the multiscale properties of GC, Mai et al. [34] proposed a representative learning model called Space2Vec, which encodes the absolute position and spatial relationship of a place. Zhao et al. [35] proposed a sensory-motor-integrated network model (SeMINet), which converts the mixed flow of sensory and motor information into a self-localization memory state and constructs a map representation of the environment. However, this model does not include an HDC or GC module, and the brain-inspired mechanism is not perfect. In the process of autonomous navigation, closed-loop detection (LCD) is essential to enable an intelligent body to reposition itself and correct accumulated errors. Using the hybrid LCD method of CNN features and the fly local-sensitive hash algorithm [36], spatial cognition can be performed in challenging large-scale environments. This method is more robust and efficient than LCD methods for existing brain-inspired navigation systems. DL mimics the hierarchical architecture of information processing in the brain by increasing the number of network layers to enhance feature extraction from the original sensor data, thereby taking advantage of the brain’s hierarchical information processing. Therefore, the DL network can perform brain-inspired spatial cognition and can be further improved not only by using a single navigation cell, such as in a location-based cell localization model, but also by the coordination of multiple navigation cell populations to expand to a more complete brain-inspired spatial cognition model.

### CANN model

Willshaw et al. [37] first proposed the concept of attractors, which refers to a nonresting steady state autonomously maintained by a dynamic system without information input. A CANN is a recursive neural network and a special type of neural dynamics model that constructs a continuous state subspace for attractors to smoothly track external inputs and achieve adaptive balance. It characterizes the encoding process of the brain for continuous variables such as orientation, motion direction, and spatial position. It is commonly used to achieve brain-inspired functions, such as encoding, storage, arithmetic, and information exchange, and is one of the few regularized neural computing models that has been experimentally validated and widely used [38].

Navigation tasks result from the encoding and representation of multilevel information in neural networks. A CANN describes the basic characteristics of brain-information-processing mechanisms and is a typical computational model for neural information representation. Its internal neuronal cluster discharge activity can encode information using an efficient, stable, and smooth parameterized memory. This is compatible with the navigation parameters of the mammalian brain navigation cell cluster emission features of animal navigation. The traditional DL model requires long training time and high computational occupancy. Inspired by the fruit fly olfactory neural circuit, Chancán et al. [39] proposed the FlyNet compact neural network and combined it with a CANN as a 1D time model to form a new compact and high-performance spatial recognition model, which achieved good localization results and was evaluated on 2 benchmark real datasets with minor viewpoint changes and extreme environmental variations, with the area under the curve result of 87% and higher speed than other models.

Path integration can be described as the displacement between the current and initial positions calculated using the model. Burak and Fiete [40] proposed a CANN GC precise path integration model that maintains accuracy close to that of rat GC path integration observed in neurophysiology. The well-known theoretical models for grid unit space discharge activity proposed currently include the CANN model and the velocity-controlled oscillation (VCO) model. When performing path integration, the discharge activity of the CANN model is affected by asymmetric interactions, and its intensity reflects the motion speed. The VCO model checks the algorithm-level description of path integration, where the path integration distance is equal to the distance traveled in a specific direction, encoded by the phase of VCO relative to the baseline frequency. When moving in a large-scale space, the distance traveled is greater than the size of the space, and path integration generates distance ambiguity. Gu et al. [41] used carrier ambiguity to solve the problem of periodic ambiguity during navigation, which is suitable for multiscale GC-like brain navigation in large spaces. Their simulation results show that the method can solve the path integration ambiguity problem in a large-scale environment of 245 m under the condition of a measurement noise of 2% with an accuracy of within 1 cm. The GC can respond to external and self-movement information and the connection between the 2 types of information during spatial cognition. Han et al. [42] combined the GC group mechanism to construct a GC group CANN model, simulating the GC response to self-movement information and enabling unmanned agents to autonomously learn and represent spaces in complex and unfamiliar environments.

Milford et al. [43] used an abstract PC and HDC to characterize the position and direction of 2D planes and proposed an attractor-based RatSLAM to construct cognitive maps. With the continuous development of intelligent navigation using navigation cells, researchers are continuously exploring higher-dimensional navigation cells. Li et al. [44] proposed a biomimetic 3D neural compass based on HDC emission characteristics to determine the spatial orientation of autonomous vehicles in a 3D environment using a CANN. Guth et al. [45] proposed an extension of the RatSLAM system based on navigation cells to the Hippo 3D algorithm, which performs simultaneous localization and mapping (SLAM) in a 3D environment to map underwater 3D environments. However, RatSLAM has not been linked to biomimetic hardware or brain-inspired algorithms, and its implementation consumes important power. Yoon and Raychowdhury [46] proposed a neuroSLAM model for an ultralow-power implementation of SLAM that uses oscillatory networks to simulate pulse networks with continuous attractor characteristics to achieve sparse energy distribution in brain-inspired spatial cognition. However, the neuroSLAM model can achieve spatial cognition only within a certain range. To integrate 3D paths for large-scale metric navigation, Yang et al. [47] used a 3D CANN to achieve path integration of self-motion information for large-scale 3D navigation and obtained accurate multiscale 3D spatial representations. However, challenges persist regarding their robustness, scalability, vulnerability, and adaptability. To overcome the dependence of manual systems on manually tuned parameters, Joseph et al. [48] introduced genetic algorithms to automatically tune these networks and utilized a multiscale CANN to improve usability and achieve fast trajectory tracking over a large range. To better enable robot navigation in large-scale environments, our institution proposes a Bayesian integration model of multisensory information, which is implemented on NeuroBayesSLAM, a monocular visual simultaneous localization and mapping system, and provides a viable Bayesian mechanism for multisensory integration, which may be applicable to other neural subsystems beyond spatial cognition in the future [49]. In addition, our institution has developed a GC computational model [50] that is based on cognitive spatial transformations for encoding and translating positions between local and global frames and provides new insights into how GCs and position cells integrate external and self-motor cues.

In general, spatial cognitive models based on CANN belong to the SLAM category. Currently, the algorithm framework based on RatSLAM focuses on introducing navigation cells from the hippocampus and medial olfactory cortex into brain-inspired navigation systems. The number of cells ranges from one to multiple, from 2D to 3D, and the sensors range from single vision to multimodal fusion. This approach constructs topological metric cognitive maps aimed at achieving low-power and highly robust autonomous brain-inspired navigation in unknown and complex environments. When applying a complete brain-based navigation cell model to actual navigation processes, practical factors, such as computational load, storage cost, and power consumption must be considered.

### SNN model

In 1997, inspired by the use of pulse signals from brain neurons to encode information, Maass [51] proposed a third-generation neural network (SNN). SNNs have strong spatiotemporal-information-processing capabilities and ultralow power consumption based on the mechanisms of brain pulse encoding and information transmission. Unlike typical neural networks, SNN neurons are not activated at each iteration. However, the membrane potential reaches a specific threshold prior to activation. When a neuron is activated, it generates a signal that is transmitted to other neurons, thereby increasing or decreasing the membrane potential. Therefore, compared with artificial neural network models, such as CANN and DL, which are closer to the actual biological structure, the output of their neurons has a pulse sequence encoded in the time dimension, and multiple neurons can achieve the ability to express a spatiotemporal 2D space.

Abstracting computational principles from the brain structure involves simulating the model as a hierarchical structure of neural processing and learning unknown environmental regions by mapping recognized environmental components to a limited number of neural representations while minimizing the learning requirements [52] to replicate brain behavior. SNN technology has surpassed the best performance of traditional DL in image classification [53], speech recognition [54], and other aspects. To improve the processing efficiency of the individual sensor information in the frequency-modulated continuous wave radar proposed by Safa [55], each sensor was processed using a biomimetic SNN with continuous-pulse spiking-timing-dependent plasticity (STDP) learning, as observed in the brain. Moreover, this system does not require an offline training stage and uses an SNN to continuously learn features from the input data through STDP. Compared with other learning-based SLAM systems, they are more robust. In the absence of sensory input, path planning can be performed by constructing a decision network that provides an intelligent agent with spatial–directional memory. The robot navigation platform Flyintel, proposed by Huang-Yu et al. [56], is inspired by the fruit fly’s ability to remember its relative direction to the target when foraging and uses an SNN to achieve basic avoidance tasks based on spatial directional memory for basic navigation. Abubaker et al. [57] utilized a customized variant of STDP to train inhibitory synapses, improve the SNN efficiency, reduce training iterations, form an evolved SNN, and enhance autonomous navigation capabilities. In addition, SNN can be combined with deep reinforcement learning (RL) to solve navigation problems in dynamic obstacles, low-visibility environments, and data transmission inaccuracies [58].

Combining the cellular mechanisms of brain navigation, Frémaux et al. [59] proposed using the SNN-based actor-critic algorithm to simulate the representation of PC positions and implemented basic navigation in the grid world of water maze simulation experiments. However, owing to the low-energy consumption characteristics of SNN computing, Xu et al. [60] utilized a hybrid framework containing SNN to implement the actor-critic algorithm’s map-free navigation scheme but did not consider scenarios with dynamic obstacles. Ramezanlou et al. [61] trained SNNs using reward-modulated, spike-temporally correlated plasticity learning methods for navigation in dynamic environments. Chen [62] used the steady-state scaling parameter of the evolutionary algorithm to optimize the time-dependent plasticity of the evolutionary spike, and the optimized SNN can be used to simulate different brain regions related to spatial navigation. Komer et al. [63] proposed a BatSLAM system for unmanned aerial vehicle navigation that performs neural morphological spatial inference in a 3D environment and utilizes an SNN to autonomously perform complex navigation tasks using only local information, and their benchmarking results showed that the system had a 97.2% success rate in indoor target navigation.

With the rise of brain-inspired computing, there have been more attempts in the current research on SNN-based brain-inspired navigation algorithms based on spatial cognition, obstacle avoidance, and path planning.

### Goal-Based Navigation

**Fig. 5.**
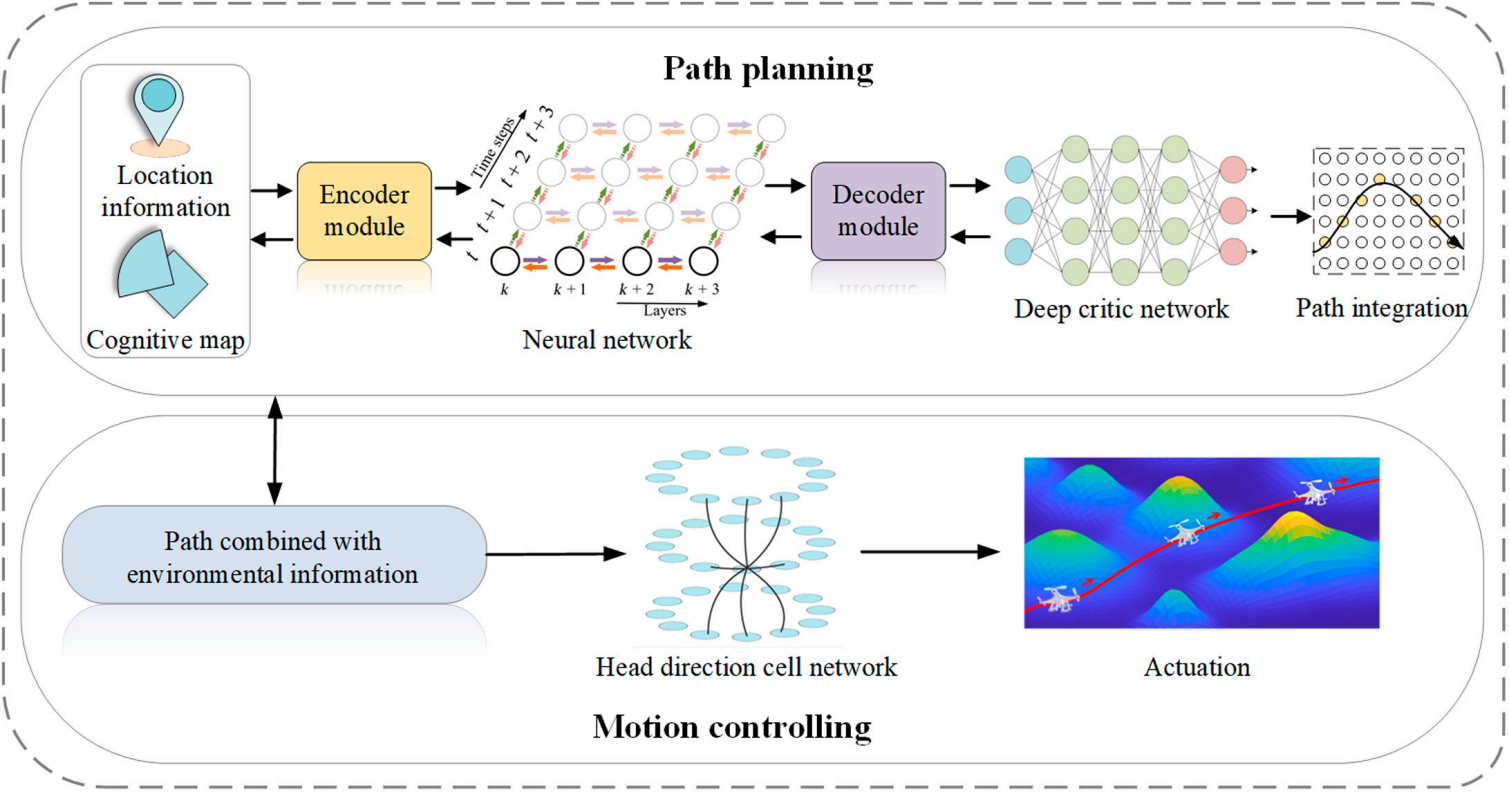
Goal-based navigation model.

Navigation refers to the monitoring and control of an object’s movement from one location to another. Autonomous navigation refers to a method that uses navigation instruments that do not rely on manually set targets or information sources to guide object movement. In known environments, animals can rapidly plan goal-based trajectories based on the observed target locations. Goal-based navigation draws on the activation mechanism of dopamine neurons and AC connections when animals navigate using cognitive maps; the main feature is the firing behavior of PCs in specific environments. In unknown complex environments, navigation algorithms must refine the functions of spatially representative cells and enhance the information interaction capability between model cells to realize navigation-position-oriented path planning and action decision-making. Location information and cognitive maps play an important role in brain-inspired navigation. In the process of sensing environmental information, the sensors commonly used in mobile robots include vision sensors, LiDAR sensors, force/contact sensors, ultrasonic sensors, robotic odometer sensors, digital magnetic compasses, and laser ranging sensors, which are used to collect sensor information, obtain location information, and construct cognitive maps. The goal-based navigation model is shown in Fig. 5. After environmental perception and spatial cognition, the obtained location information and cognitive map are encoded and decoded and then iteratively processed by a deep critic network to complete path planning. By combining the path and environmental information, obstacle avoidance and safe movement toward the destination can be achieved. The primary tasks of goal-based navigation include path planning and obstacle avoidance.

### Supervised learning goal-based navigation

Supervision-based learning mechanisms can be categorized as supervised or unsupervised.

Supervised learning is a machine learning problem that learns predictive models from labeled data. Supervised learning models can be applied to environmental detection during the navigation process and to overall target decision-making and path planning. Traditional models are suitable for path-planning algorithms such as probabilistic roadmaps and fast-exploration random trees. However, these algorithms ignore large amounts of visual information and are highly sensitive to the noise in robot odometry and sensor data. Watkins-Valls et al. [64] proposed a target-driven visual navigation that does not require maps, azimuth, or odometry. To determine whether a Done action should be proposed, other action decisions should be made only when the network determines that a Done action should not be proposed. Brown et al. [65] proposed uncertainty modulation learning, a lifelong learning algorithm based on neural modulation that combines uncertainty measurements and self-supervision mechanisms to enhance navigation through self-supervision.

Unsupervised learning refers to solving various problems in pattern recognition based on training samples with unknown (unlabeled) categories when it is difficult to manually label categories or when the cost of manually labeling categories is too high in the absence of sufficient prior knowledge. Using GC to encode the current and target positions separately in multiscale space and establish specific connections between the current and target differences and motor neurons through competitive learning, the robot achieves continuous target-guided navigation [66], and the hippocampus, prefrontal cortex, and amygdala models form a brain-inspired neural network model [67], which obtains specific knowledge from a limited amount of experience through training and inference to determine navigation paths. Target-oriented navigation based on an unsupervised learning mechanism has the advantages of autonomous learning, navigation behavior decision-making, and higher flexibility. However, its disadvantages include low learning efficiency, less integration with brain-inspired mechanisms, and incomplete brain-inspired mechanisms.

### Deep RL-based goal navigation

RL is a paradigm and methodology used to describe and solve the problem of agents maximizing returns or achieving specific goals through learning strategies during their interactions with the environment. Deep RL (DRL) combines DL and RL, while possessing both DL perception and RL decision-making abilities, resulting in a stronger ability to solve practical problems. DRL target navigation accumulates rewards, optimizes behavior strategies, achieves better path planning, and completes the navigation of the location load environment through a continuous process between the agent and the environment.

On the basis of DRL target navigation, the DL and RL algorithms were combined. Zhang et al. [68] combined short-term memory and progress optimization algorithms into a local visual navigation model and designed a new reward function. The target is trained through factors such as the movement of the mobile robot, distance between the robot and the target, and the running time of the robot. Targeted navigation in large and complex scenes can be achieved without the need for maps or human intervention, and intelligent agents use learning methods that map environmental states to behaviors to improve computational efficiency. Path-planning and behavior decision-making algorithms include many aspects. The Deep Deterministic Policy Gradient guides agents on how to interact with the surrounding environment, which can effectively complete path planning tasks in complex continuous environments [69]. In addition, utilizing known experiences and environmental memory, Ma et al. [70] proposed a DRL model with a unique long-term memory ability based on recurrent neural networks and continuous historical states as inputs to achieve target navigation in a dynamic environment without maps.

In addition to combining DL and RL, combinations of RL with imitation and incremental learning can achieve target-oriented navigation. Li [71] combined imitation learning with RL to enhance navigational intelligence in urban autonomous driving scenarios. The embedded actor-critic architecture proposed by Rao et al. [72] introduced an inverse dynamic model and a multiobjective collaborative model for multiobjective visual navigation tasks, whereas Campari et al. [73] utilized abstract models of finite-state machines to increase unknown environments. The improved incremental brain development model proposed by Luo and Fang [74] performs incremental learning on environmental temporal data and has better real-time computational performance, meeting the requirements of mobile robots in processing complex scene classification tasks for better self-localization and navigation decision-making.

Inspired by the brain navigation mechanism, Hu et al. [75] proposed a hierarchical model based on position cells and GCs, combined with *Q* value redundancy during RL training, which has been used in the Morris water maze experiment to navigate the state-action-reward-state-action (SARSA) algorithm and achieve the selection of target paths. Huang et al. [76] separated perception and control and utilized multimodal perception and reliable online interaction with the surrounding environment to achieve direct policy learning, which can generate flexible actions and achieve smooth and safe navigation in complex environments. They further proposed a novel DRL system for autonomous navigation of mobile robots [77], which includes 3 modules: map navigation, multiview perception, and multibranch control. The multiview perception module combines the attention mechanism to filter out redundant information caused by multicamera perception, and the system can achieve a good balance between global navigation and local path planning, ultimately achieving its goal. Liu et al. [78] combined the interaction between GCs and positional cells with the environment in a new navigation framework and combined environmental experience with RL to achieve rapid and high-precision construction of predictive maps and path planning. However, in the current DRL-based target navigation models, which combine brain navigation mechanisms, the focus is primarily on information-processing processes that achieve the interaction between GCs and position cells with the environment. To adapt these models to large-scale tasks, more sophisticated mechanisms need to be imitated, such as the incorporation of head-facing cells into the navigation model.

Existing implementations of DRL-based target navigation models have shortcomings, such as a low navigation success rate and efficiency, and there are few references to animal brain navigation mechanisms. DRL also lacks integration of physiological mechanisms for animal-oriented target navigation decision-making processes.

### Obstacle avoidance in goal-based navigation

During navigation tasks in unknown environments, intelligent agents must recognize and avoid obstacles. The necessary condition for achieving obstacle avoidance is environmental perception, which involves obtaining information about the unknown surrounding environment through sensors, including the size, shape, and position of obstacles. Sensor technology plays an important role in navigating and avoiding obstacles. The sensors used for obstacle detection include ultrasonic sensors. To collect and process data, Singh et al. [79] used Savitzky–Golay filters, including infrared sensors, monocular and stereo cameras, and LiDAR sensors for data smoothing. Compared to visual sensors, LiDAR sensors can actively project lasers, which can more accurately understand the distance and depth information of obstacles. Moreover, they were not significantly affected by changes in environmental lighting. Because of the high accuracy and stability of LiDAR sensors, the overall performance of brain-inspired navigation SLAM systems can be further improved [80].

After identifying obstacles, obstacle avoidance algorithms are used to replicate the navigation path and achieve safe and efficient navigation. DL models play an important role in research on the obstacle avoidance algorithm. After obtaining sensing information, Aishwarya and Panda [81] used conditional GANs (CGANs) to separate road boundaries, generate images of the obtained data, and mark roads in red. The labeled data were used to train CGANs, which can still recognize road boundaries under poor lighting conditions at night. In crowded environments prone to collisions, Shan et al. [82] designed a trajectory planner based on DRL, which provides assurance for intelligent agents to navigate through complex static environments during the navigation process. When combined with other strategy control algorithms, such as learning-based nonlinear model preinstruction control [83], it enhances perception and decision-making, enabling obstacle avoidance in unstructured environments. Ruan et al. [84] proposed the LN-D3QN algorithm, whereas Phueakthong et al. [85] selected depth-deterministic policy gradients that can operate in a continuous action space; a mobile robot utilizing this strategy can navigate a target in an unknown environment with a minimum success rate of 69.7%. Both achieved collision-free autonomous navigation for mobile robots and demonstrated the ability to quickly adapt to unknown environments, efficiently completing obstacle avoidance navigation tasks within the LN-D3QN network structure. However, relying solely on static obstacle avoidance proves insufficient when dynamic obstacles appear. Thus, dynamic obstacle avoidance should be considered. In other words, after predicting the dynamic obstacles, local path planning should be used during path tracking [86], and the 2 types of algorithms should pass through numerous static and dynamic obstacles.

Brain-inspired computing technology provides new ideas for navigational obstacle avoidance, implementing a fault-tolerant solution for human cognitive function through neural morphology simulation and implementing a new self-learning SNN model with scalability [87]. Xing et al. [88] used an SNN to simulate obstacle avoidance movements in multiple brain regions, including 3 modules that simulate the perceptual function of the visual cortex, obstacle avoidance function of the cerebellum, and prediction and planning function of the prefrontal cortex. Hyperdimensional computing is a relatively new computing mode that uses large vectors (such as 10,000 bits each) for computation and is inspired by the neural activity patterns of the human brain [89]. It uses high-capacity hypervectors to represent navigation tasks and prioritizes behaviors with potential in resource-constrained systems. In addition, brain–computer interfaces (BCIs) also provide directions for the brain-inspired navigation of mobile robots. Li et al. [90] proposed a road segmentation method based on centroidal Voronoi tessellation, which utilizes a BCI that fuses human and robot intelligence to generate selectable navigation targets in a road area. It also selects an arbitrary target using an autonomous navigation function that can reach human-selected targets for obstacle avoidance.

## Discussion and Conclusion

Researchers have studied brain-inspired navigation in unknown and complex environments from 3 perspectives: brain-inspired environment perception, brain-inspired spatial cognition, and target-oriented navigation, forming a research system that integrates mechanism analysis, modeling, simulation, and experimental verification. Table presents the results.

**Table.** List of reported brain-inspired technology for mobile robots

Authors	Cells	Dimension	Sensor	Network	References
Li et al.	PC	2D	Monocular camera	RBFNN	[28]
Walch et al.	–	2D\3D	RGB-D sensors, camera	CNN, LSTM	[30]
Sindhu and Saravanan	–	3D	–	GAN	[32]
Mai et al.	GC	2D	LiDAR	Encoder–decoder	[34]
Zhao et al.	PC, GC, SC, HDC	3D	Monocular camera	SeMINet	[35]
Liu et al.	PC, HDC, GC, SC, DC	3D	MEMS, DTMB	CANN	[27]
RatSLAM	PC, HDC	2D	Monocular camera	CANN	[43]
Li et al.	HDC	3D	IMU	CANN	[44]
Han et al.	PC, GC	2D	–	CANN	[42]
Li et al.	HDC	3D	IMU	CANN	[44]
Gu et al.	GC	2D	GNSS	CANN, VCO	[41]
Yoon and Raychowdhury	PC, HDC	2D	Monocular camera	SNN	[46]
Dong et al.	PC, GC	2D	Monocular camera	SNN	[54]
Komer et al.	GC, HDC	3D	RGB-D camera	SNN	[63]
Xing et al.	GC, PC	3D	Monocular camera	SNN	[88]
Edvardsen et al.	GC	2D	Camera	ANN	[66]
Mizutani et al.	PC	3D	Camera	BNN	[67]
Luo et al.	Nerve Cell	3D	–	SOINN	[74]
Hao et al.	GC, PC	3D	Camera	DQN	[75]
Liu et al.	GC, PC	2D\3D	Wheel odometer, camera	–	[78]
Aishwarya et al.	–	2D\3D	3D LiDAR	CGAN	[81]
Ruan et al.	–	3D	Camera	D3QN	[84]
Yang et al.	Nerve cell	3D	Radar sensors,camera, IoVs	SNN	[87]

In brain-inspired environmental perception, environmental information is obtained through multiple senses. Presently, sensors with multiple modes are used to obtain information such as images, audio, dynamics, light waves, and point clouds. A single mode can be processed or integrated into multimodal information to achieve more comprehensive environmental perception. Currently, research on multimodal-information-fusion-processing mechanisms is underway, and a more comprehensive and efficient information fusion extraction method has not yet been developed. In the future, for autonomous navigation in unknown environments, more sensor modes can be integrated, including not only vision and touch but also hearing and temperature, in certain special environments to achieve more control functions, such as area search and object search, and improve fault safety mechanisms. In addition, the sensor end can be integrated with a certain amount of computing power to achieve lower and higher efficiencies. Many of the current processing algorithms lack dynamism. Future developments can integrate dynamic information into models to better support sensor information processing, adapt to complex environmental switching, and become more intelligent.

When conducting brain-inspired spatial cognition, information obtained from environmental perception is utilized to achieve spatial information cognition. Environmental cognitive maps are constructed using DL networks, CANN models, and SNN models to simulate the navigation mechanism of animal brains. However, there is currently a lack of integration between various types of models and biological mechanisms inspired not only by a single navigation cell but also by many localization models based on location cells. In the future, multiple navigational cell populations can be synergistically coordinated to develop more complete brain-inspired spatial cognitive models. Better integration of the biological mechanisms of animal brain navigation into various types of patterns, achieving brain-inspired spatial cognition, and enhancing the generalizability of models such that they can be applied to different scenarios are urgent problems that need to be solved. Simultaneously, when applying a complete brain-based navigation cell model to actual navigation processes, practical factors such as computational load, storage cost, and power consumption must be considered.

In the process of achieving target-oriented navigation, supervised learning and DRL are used to utilize the cognitive map formed by spatial cognition for path planning and action decision making at the target locations. Existing models have the drawbacks of low navigation success rate and efficiency, particularly in obstacle avoidance tasks, where animal brain navigation mechanisms are less utilized and models such as brain mechanisms are not perfect enough. For example, perception and spatial memory systems can be combined into navigation models. This also requires researchers to continue exploring methods to integrate biological mechanisms into target navigation and obstacle avoidance, while improving the navigation success rate and efficiency.

In addition, brain-inspired navigation for unknown and complex environments can be successfully performed by a single intelligent body. However, for large and complex navigation tasks, relying on a single intelligent body is insufficient. Thus, it becomes necessary to gather multiple intelligent bodies and form a cluster of intelligent bodies to realize large and complex tasks. Consequently, brain-inspired navigation technology can be applied to large-scale distributed intelligent body cluster navigation in the future. Brain-inspired navigation technology has a wide range of prospective applications in mobile robotics. For example, when multiple emergency rescue robot swarms perform search-and-rescue tasks, brain-inspired navigation technology can be used to explore and model unknown environments and construct incremental maps. Such exploratory modeling can help robots understand the environment better and play a key role in identifying unknown targets. Through cognitive capabilities based on brain-inspired intelligence, robots can determine the attributes of unknown targets, thus improving the efficiency of search and rescue. The application of brain-inspired intelligence allows multiple robots to work together in unknown and complex environments, respond quickly to emergencies, and provide more effective rescue services. In addition, in industrial scenarios, mobile robots can use brain-inspired navigation technology to carry out intelligent inspection and monitoring, identify abnormalities, and take corresponding measures in a timely manner to improve the safety and stability of equipment operation; in the field of intelligent transport and logistics, mobile robots can achieve autonomous planning of paths, avoid traffic congestion, and efficiently complete the task of transporting goods and other tasks using brain-inspired navigation technology to enhance the intelligence level of the transport and logistics system. In the field of intelligent transport and logistics, mobile robots can achieve autonomous path planning, avoid traffic congestion, efficiently complete cargo transport, and perform other tasks through brain-inspired navigation technology, thereby improving the intelligence level of transport and logistics systems. In conclusion, brain-inspired navigation technology provides mobile robots with intelligent cognitive and decision-making capabilities, enabling them to perform various tasks more flexibly and efficiently in complex and dynamic environments that are convenient and secure for human production and life.

To achieve more efficient brain-inspired navigation, it is necessary to draw inspiration from the biological mechanisms of efficient animal brain navigation in processes such as environmental perception, spatial cognition, and target navigation and introduce them into the model to complete large-scale spatial environment navigation. Existing models have shortcomings in terms of the navigation success rate and efficiency, and there are few references to animal brain navigation mechanisms. Researchers should continue to explore methods for integrating biological mechanisms into targeted navigation. Brain-inspired navigation technology is a common challenge facing the next generation of intelligent navigation in interdisciplinary fields such as computer science, brain and neuroscience, cognitive science, navigation and control, robotics, and artificial intelligence. Interdisciplinary scholars must engage in communication and cooperation, inspire each other, jointly promote technological development, strive to propose a new generation of neural network methods, imitate the brain at the structural level, approach the brain at the device level, and surpass the brain at the intelligence level to achieve low power consumption, high robustness, self-learning, and self-evolution in brain-inspired autonomous intelligent navigation.

## Data Availability

Data sharing is not applicable to this article, as no new data were created or analyzed in this study.

## References

[1] Honkanen A, Adden A, Da Silva Freitas J, Heinze S. The insect central complex and the neural basis of navigational strategies. J Exp Biol. 2019;222(Suppl_1):jeb188854. 30728235 10.1242/jeb.188854 PMC6474401

[2] O’Keefe J, Dostrovsky J. The hippocampus as a spatial map: Preliminary evidence from unit activity in the freely-moving rat. *Brain Res*. 1971;34:171–175. 5124915 10.1016/0006-8993(71)90358-1

[3] Hori E, Nishio Y, Kazui K, Umeno K, Tabuchi E, Sasaki K, Endo S, Ono T, Nishijo H. Place-related neural responses in the monkey hippocampal formation in a virtual space. Hippocampus. 2005;15(8):991–996. 16108028 10.1002/hipo.20108

[4] Guy M. Neurosurgical recordings reveal cellular networks underlying human spatial navigation. Neurosurgery. 2004;3:1.

[5] Thompson LT, Best PJ. Long-term stability of the place-field activity of single units recorded from the dorsal hippocampus of freely behaving rats. Brain Res. 1990;509(2):299–308. 2322825 10.1016/0006-8993(90)90555-p

[6] Muller R, Kubie J. The effects of changes in the environment on the spatial firing of hippocampal complex-spike cells. J Neurosci. 1987;7(7):1951–1968. 3612226 10.1523/JNEUROSCI.07-07-01951.1987 PMC6568940

[7] Leutgeb S, Leutgeb JK, Barnes CA, Moser EI, McNaughton BL, Moser M-B. Independent codes for spatial and episodic memory in hippocampal neuronal ensembles. Science. 2005;309(5734):619–623. 16040709 10.1126/science.1114037

[8] Killian NJ, Jutras MJ, Buffalo EA. A map of visual space in the primate entorhinal cortex. Nature. 2012;491(7426):761–764. 23103863 10.1038/nature11587 PMC3565234

[9] Jacobs J, Weidemann CT, Miller JF, Solway A, Burke JF, Wei XX, Suthana N, Sperling MR, Sharan AD, Fried I, et al. Direct recordings of grid-like neuronal activity in human spatial navigation. Nat Neurosci. 2013;16(9):1188–1190. 23912946 10.1038/nn.3466 PMC3767317

[10] Mouritsen H. Long-distance navigation and magnetoreception in migratory animals. Nature. 2018;558:50–59. 29875486 10.1038/s41586-018-0176-1

[11] Towse BW, Barry C, Bush D, Burgess N. Optimal configurations of spatial scale for grid cell firing under noise and uncertainty. Philos Trans R Soc B. 2014;369(1635):20130290. 10.1098/rstb.2013.0290 PMC3866454 24366144

[12] Taube J, Muller R, Ranck J. Head-direction cells recorded from the postsubiculum in freely moving rats. I. Description and quantitative analysis. J Neurosci. 1990;10(2):420–435. 2303851 10.1523/JNEUROSCI.10-02-00420.1990 PMC6570151

[13] Kim M, Maguire EA. Encoding of 3D head direction information in the human brain. Hippocampus. 2019;29(7):619–629. 30561118 10.1002/hipo.23060 PMC6618148

[14] Taube J, Muller R, Ranck J. Head-direction cells recorded from the postsubiculum in freely moving rats. II. Effects of environmental manipulations. J Neurosci. 1990;10(2):436–447. 2303852 10.1523/JNEUROSCI.10-02-00436.1990 PMC6570161

[15] Park S, Lee K, Song H, Cho J, Park S-Y, Yoon E. Low-power, bio-inspired time-stamp-based 2-D optic flow sensor for artificial compound eyes of micro air vehicles. IEEE Sensors J. 2019;19(24):12059–12068.

[16] Chiu M-Y, Chen GC, Hsu TH, Liu RS, Lo CC, Tang KT, Chang MF, Hsieh CC. A multimode vision sensor with temporal contrast pixel and column-parallel local binary pattern extraction for dynamic depth sensing using stereo vision. IEEE J Solid State Circuits. 2023;58(10):2767–2777.

[17] Shukla R, Routray PK, Tiwari K, LaValle SM, Manivannan M. Monofilament whisker-based mobile robot navigation. Paper presented at: 2021 IEEE World Haptics Conference (WHC); 2021 July 06–09; Montreal, QC, Canada.

[18] Weerakkodi Mudalige ND, Nazarova E, Babataev I, Babataev P, Fedoseev A, Cabrera MA, Tsetserukou D. DogTouch: CNN-based recognition of surface textures by quadruped robot with high density tactile sensors. Paper presented at: 2022 IEEE 95th Vehicular Technology Conference: (VTC2022-Spring); 2022 June 19–22; Helsinki, Finland.

[19] Borkar N, Krishnamurthy P, Tzes A, Khorrami F. Autonomous navigation of quadrotors using tactile feedback. Paper presented at: 2023 9th International Conference on Automation, Robotics and Applications (ICARA); 2023 February 10–12; Abu Dhabi, United Arab Emirates.

[20] Weerakoon K, Sathyamoorthy AJ, Liang J, Guan T, Patel U, Manocha D. GrASPE: Graph based multimodal fusion for robot navigation in outdoor environments. IEEE Robot Autom Lett. 2023;8(12):8090–8097.

[21] Linegar C, Churchill W, Newman P. Made to measure: Bespoke landmarks for 24-hour, all-weather localisation with a camera. Paper presented at: 2016 IEEE International Conference on Robotics and Automation (ICRA); 2016 May 16–21; Stockholm, Sweden.

[22] Chengqing W, Chenning L, Haowei X. An improved visual indoor navigation method based on fully convolutional neural network. Paper presented at: 2020 IEEE International Conference on Signal Processing, Communications and Computing (ICSPCC); 2020 August 21–24; Macau, China.

[23] Sun C, Qiao N, Sun J. Robust feature matching based on adaptive ORB for vision-based robot navigation. Paper presented at: 2021 36th Youth Academic Annual Conference of Chinese Association of Automation (YAC); 2021 May 28–30; Nanchang, China.

[24] Wang H, Huang L, Du C, Li D, Wang B, He H. Neural encoding for human visual cortex with deep neural networks learning ‘what’ and ‘where. IEEE Trans Cogn Dev Syst. 2021;13(4):827–840.

[25] Plebe A, Kooij JFP, Pietro Rosati Papini G, Da Lio M. Occupancy grid mapping with cognitive plausibility for autonomous driving applications. Paper presented at: 2021 IEEE/CVF International Conference on Computer Vision Workshops (ICCVW); 2021 October 11–17; Montreal, BC, Canada.

[26] Fazeli N, Oller M, Wu J, Wu Z, Tenenbaum JB, Rodriguez A. See, feel, act: Hierarchical learning for complex manipulation skills with multisensory fusion. Sci Robot. 2019;4(26):eaav3123. 33137764 10.1126/scirobotics.aav3123

[27] Liu X, Chen L, Jiao Z, Yu F, Lu X, Liu Z, Ruan Y. A neuro-inspired positioning system integrating MEMS sensors and DTMB signals. IEEE Trans Broadcast. 2023;69(3):823–831.

[28] Li W, Wu D, Dai C, Wu D. A position representation method based on the localization mechanism of rat hippocampus. Paper presented at: 2022 5th International Conference on Artificial Intelligence and Big Data (ICAIBD); 2022 May 27–30; Chengdu, China.

[29] Li X, Guo K, Jia T. Visual perception and navigation of security robot based on deep learning. Paper presented at: 2020 IEEE International Conference on Mechatronics and Automation (ICMA); 2020 October 13–16; Beijing, China.

[30] Walch F, Hazirbas C, Leal-Taixé L, Sattler T, Hilsenbeck S, Cremers D. Image-based localization using LSTMs for structured feature correlation. Paper presented at: 2017 IEEE International Conference on Computer Vision (ICCV); 2017 October 22–29; Venice, Italy.

[31] Yang C, Liu Y, Zell A. RCPNet: Deep-learning based relative camera pose estimation for UAVs. Paper presented at: 2020 International Conference on Unmanned Aircraft Systems (ICUAS); 2020 October 1–4; Athens, Greece.

[32] Sindhu S, Saravanan M. Part-based convolutional neural network and dual interactive Wasserstein generative adversarial networks for land mark detection and localization of autonomous robots in outdoor environment. Paper presented at: 2022 1st International Conference on Computational Science and Technology (ICCST); 2022 November 9–10; Chennai, India.

[33] Zaman A, Yangyu F, Ayub MS, Guoyun L, Shiva L. Brain inspired keypoint matching for 3D scene reconstruction. Paper presented at: 2022 8th international conference on virtual reality (ICVR); 2022 May 26–28; Nanjing, China.

[34] Mai G, Janowicz K, Yan B. Multi-scale representation learning for spatial feature distributions using grid cells. ArXiv. 2020. 10.48550/arXiv.2003.00824.

[35] Zhao D, Zhang Z, Lu H, Cheng S, Si B, Feng X. Learning cognitive map representations for navigation by sensory–motor integration. IEEE Trans Cybern. 2022;52(1):508–521. 32275629 10.1109/TCYB.2020.2977999

[36] Li J, Tang H, Yan R. A hybrid loop closure detection method based on brain-inspired models. IEEE Trans Cogn Dev Syst. 2022;14(4):1532–1543.

[37] Willshaw DJ, Dayan P, Morris RGM. Memory, modelling and Marr: a commentary on Marr (1971) ‘Simple memory: A theory of archicortex’. Philos Trans R Soc B. 2015;370(1666):20140383. 10.1098/rstb.2014.0383 PMC4360131 25750246

[38] Wu S, Wong KYM, Fung CCA. Continuous attractor neural networks: Candidate of a canonical model for neural information representation [version 1; peer review: 2 approved]. F1000Research. 2016;5(F1000 Faculty Rev):156. 10.12688/f1000research.7387.1 PMC4752021 26937278

[39] Chancán M, Hernandez-Nunez L, Narendra A, Barron AB, Milford M. A hybrid compact neural architecture for visual place recognition. IEEE Rob Autom Lett. 2020;5(2):993–1000.

[40] Burak Y, Fiete IR. Accurate path integration in continuous attractor network models of grid cells. PLoS Comput Biol. 2009;5(2): Article e1000291. 19229307 10.1371/journal.pcbi.1000291 PMC2632741

[41] Gu Y, Zhao X, Dai C, Zhang L. Research on ambiguity calculation method of brain-like grid cell path integration under large-scale conditions. Paper presented at: 2020 International Conference on Robots & Intelligent System (ICRIS); 2020 November 7–8; Sanya, China.

[42] Han K, Wu D, Lai L, He J. The self-motion information response model in brain-inspired navigation. IEEE Access. 2020;8:49717–49729.

[43] Milford MJ, Wyeth GF, Prasser DP. RatSLAM: A hippocampal model for simultaneous localisation and mapping. Paper presented at: IEEE International Conference on Robotics and Automation, 2004; 2004 April 26–May 1; New Orleans, LA, USA.

[44] Li B, Liu Y, Lai L. A bio-inspired 3-D neural compass based on head direction cells. IEEE Access. 2021;9:110753–110761.

[45] Guth FA, Silveira L, Amaral M, Botelho S, Drews P. Underwater visual 3D SLAM using a bio-inspired system. Paper presented at: 2013 Symposium on Computing and Automation for Offshore Shipbuilding; 2013 March 14–15; Rio Grande, Brazil.

[46] Yoon J-H, Raychowdhury A. NeuroSLAM: A 65-nm 7.25-to-8.79-TOPS/W mixed-signal oscillator-based SLAM accelerator for edge robotics. IEEE J Solid State Circuits. 2021;56(1):66–78.

[47] Yang C, Xiong Z, Liu J, Chao L, Chen Y. A path integration approach based on multiscale grid cells for large-scale navigation. IEEE Trans Cogn Dev Syst. 2022;14(3):1009–1020.

[48] Joseph T, Fischer T, Milford M. Trajectory tracking via multiscale continuous attractor networks. Paper presented at: 2023 IEEE/RSJ International Conference on Intelligent Robots and Systems (IROS); 2023 October 1–5; Detroit, MI, USA.

[49] Zeng T, Tang F, Ji D, Si B. NeuroBayesSLAM: Neurobiologically inspired Bayesian integration of multisensory information for robot navigation. Neural Netw. 2020;126:21–35. 32179391 10.1016/j.neunet.2020.02.023

[50] Zhang Z, Tang F, Li Y, Feng X. Modeling the grid cell activity based on cognitive space transformation. Cogn Neurodynamics. 2023;18:1–17. 10.1007/s11571-023-09972-w PMC11143158 38826659

[51] Maass W. Networks of spiking neurons: The third generation of neural network models. Neural Netw. 1997;10(9):1659–1671.

[52] Tang G, Michmizos KP. Gridbot: An autonomous robot controlled by a spiking neural network mimicking the brain’s navigational system. In: *Proceedings of the international conference on neuromorphic systems*. Association for Computing Machinery; 2018. p. 1–8.

[53] Zhang W, Li P. Spike-train level backpropagation for training deep recurrent spiking neural networks. In: *Advances in neural information processing systems*. MIT Press; 2019. p. 32.

[54] Dong M, Huang X, Xu B. Unsupervised speech recognition through spike-timing-dependent plasticity in a convolutional spiking neural network. PLoS One. 2018;13(11): Article e0204596. 30496179 10.1371/journal.pone.0204596 PMC6264808

[55] Safa A. Fusing event-based camera and radar for SLAM using spiking neural networks with continual STDP learning. Paper presented at: 2023 IEEE International Conference on Robotics and Automation (ICRA); 2023 May 29–June 2; London, UK.

[56] Huang-Yu Y, Huang HP, Huang YC. Flyintel – A platform for robot navigation based on a brain-inspired spiking neural network. Paper presented at: 2019 IEEE International Conference on Artificial Intelligence Circuits and Systems (AICAS); 2019 May 18–20; Hsinchu, Taiwan.

[57] Abubaker BA, Ahmed SR, Guron AT, Fadhil M, Algburi S, Abdulrahman BF. Spiking neural network for enhanced mobile robots’ navigation control. Paper presented at: 2023 7th International Symposium on Innovative Approaches in Smart Technologies (ISAS); 2023 November 23–25; Istanbul, Turkiye.

[58] Yang B, Yuan M, Zhang C, Hong C, Pan G, Tang H. Spiking reinforcement learning with memory ability for mapless navigation. Paper presented at: 2023 IEEE/RSJ International Conference on Intelligent Robots and Systems (IROS); 2023 October 1–5; Detroit, MI, USA.

[59] Frémaux N, Sprekeler H, Gerstner W, Sprekeler H, Gerstner W. Reinforcement learning using a continuous time actor-critic framework with spiking neurons. PLoS Comput Biol. 2013;9(4): Article e1003024. 23592970 10.1371/journal.pcbi.1003024 PMC3623741

[60] Xu R, Wu Y, Qin X, Zhao P. Population-coded spiking neural network with reinforcement learning for mapless navigation. Paper presented at: 2022 International Conference on Cyber-Physical Social Intelligence (ICCSI); 2022 November 18–21; Nanjing, China.

[61] Ramezanlou MT, Azimirad V, Sotubadi SV, Janabi-Sharifi F. Spiking neural controller for autonomous robot navigation in dynamic environments. Paper presented at: 2020 10th International Conference on Computer and Knowledge Engineering (ICCKE); 2020 October 29–30; Mashhad, Iran.

[62] Chen K. Differential spatial representations in hippocampal CA1 and subiculum emerge in evolved spiking neural networks. Paper presented at: 2021 International Joint Conference on Neural Networks (IJCNN); 2021 July 18–22; Shenzhen, China.

[63] Komer B, Jaworski P, Harbour S, Eliasmith C, DeWolf T. BatSLAM: Neuromorphic spatial reasoning in 3D environments. Paper presented at: 2022 IEEE/AIAA 41st Digital Avionics Systems Conference (DASC); 2022 September 18–22; Portsmouth, VA, USA.

[64] Watkins-Valls D, Xu J, Waytowich N, Allen P. Learning your way without map or compass: Panoramic target driven visual navigation. Paper presented at: 2020 IEEE/RSJ International Conference on Intelligent Robots and Systems (IROS); 2020 October 24–January 24; Las Vegas, NV, USA.

[65] Brown R, Brna A, Cook J, Park S, Aguilar-Simon M. Uncertainty-driven control for a self-supervised lifelong learning drone. Paper presented at: IGARSS 2022-2022 IEEE International Geoscience and Remote Sensing Symposium; 2022 July 17–22; Kuala Lumpur, Malaysia.

[66] Edvardsen V. Goal-directed navigation based on path integration and decoding of grid cells in an artificial neural network. Nat Comput. 2019;18(1):13–27.

[67] Mizutani A, Tanaka Y, Tamukoh H, Katori Y, Tateno K, Morie T. Brain-inspired neural network navigation system with hippocampus, prefrontal cortex, and amygdala functions. Paper presented at: 2021 International Symposium on Intelligent Signal Processing and Communication Systems (ISPACS); 2021 November 16–19; Hualien City, Taiwan.

[68] Zhang Y, Feng W, Yang Z, Zhou Z, Zhu Z, Wang W. Visual navigation of mobile robots in complex environments based on distributed deep reinforcement learning. Paper presented at: 2022 6th Asian Conference on Artificial Intelligence Technology (ACAIT); 2022 December 9–11; Changzhou, China.

[69] Zhang K, Hu Y, Huang D, Yin Z. Target tracking and path planning of mobile sensor based on deep reinforcement learning. Paper presented at: 2023 IEEE 12th Data Driven Control and Learning Systems Conference (DDCLS); 2023 May 12–14; Xiangtan, China.

[70] Ma H, Wang S, Zhang S, Ren S, Wang H. Map-less end-to-end navigation of mobile robots via deep reinforcement learning. Paper presented at: 2023 IEEE 13th International Conference on Electronics Information and Emergency Communication (ICEIEC); 2023 July 14–16; Beijing, China.

[71] Li Z. A hierarchical autonomous driving framework combining reinforcement learning and imitation learning. Paper presented at: 2021 International Conference on Computer Engineering and Application (ICCEA); 2021 June 25–27; Kunming, China.

[72] Rao Z, Wu Y, Yang Z, Zhang W, Lu S, Lu W, Zha ZJ. Visual navigation with multiple goals based on deep reinforcement learning. IEEE Trans Neur Netw Learn Syst. 2021;32(12):5445–5455. 10.1109/TNNLS.2021.3057424 33667168

[73] Campari T, Lamanna L, Traverso P, Serafini L, Ballan L. Online learning of reusable abstract models for object goal navigation. Paper presented at: 2022 IEEE/CVF Conference on Computer Vision and Pattern Recognition (CVPR); 2022 June 18–24; New Orleans, LA, USA.

[74] Luo H, Fang Y. A scene classification method based on improved incremental brain-like developmental model. Paper presented at: 2023 42nd Chinese Control Conference (CCC); 2023 July 24–26; Tianjin, China.

[75] Hu L, Hao K, Cai X, Chen L. A spatial cognitive cells inspired goal-directed navigation model. Paper presented at: 2019 IEEE International Conference on Artificial Intelligence and Computer Applications (ICAICA); 2019 March 29–31; Dalian, China.

[76] Huang X, Deng H, Zhang W, Song R, Li Y. Towards multi-modal perception-based navigation: A deep reinforcement learning method. IEEE Robot Autom Lett. 2021;6(3):4986–4993.

[77] Huang X, Chen W, Zhang W, Song R, Cheng J, Li Y. Autonomous multi-view navigation via deep reinforcement learning. Paper presented at: 2021 IEEE International Conference on Robotics and Automation (ICRA); 2021 May 30–June 5; Xi’an, China.

[78] Liu D, Lyu Z, Zou Q, Bian X, Cong M, Du Y. Robotic navigation based on experiences and predictive map inspired by spatial cognition. IEEE/ASME Trans Mech. 2022;27(6):4316–4326.

[79] Singh J, Dhuheir M, Refaey A, Erbad A, Mohamed A, Guizani M, Navigation and obstacle avoidance system in unknown environment. Paper presented at: 2020 IEEE Canadian Conference on Electrical and Computer Engineering (CCECE); 2020 August 30–September 02; London, ON, Canada.

[80] Zhuang G, Bing Z, Huang Y, Huang K, Knoll A. A biologically-inspired simultaneous localization and mapping system based on LiDAR sensor. Paper presented at: 2022 IEEE/RSJ International Conference on Intelligent Robots and Systems (IROS); 2022 October 23–27; Kyoto, Japan.

[81] Aishwarya LT, Panda M. Road boundary detection using 3D-to-2D transformation of LIDAR data and conditional generative adversarial networks. Paper presented at: 2020 11th International Conference on Computing, Communication and Networking Technologies (ICCCNT); 2020 July 1–3; Kharagpur, India.

[82] Shan Q, Wang W, Guo D, Sun X, Jia L. Improving the ability of robots to navigate through crowded environments safely using deep reinforcement learning. Paper presented at: 2022 International Conference on Advanced Robotics and Mechatronics (ICARM); 2022 July 9–11; China: Guilin.

[83] Doukhi O, Kang D, Ryu Y, Lee J, Lee DJ. Learning-based NMPC for agile navigation and obstacle avoidance in unstructured environments. Paper presented at: 2023 23rd International Conference on Control, Automation and Systems (ICCAS); 2023 October 17–20; Yeosu, Republic of Korea.

[84] Ruan X, Lin C, Huang J, Li Y. Obstacle avoidance navigation method for robot based on deep reinforcement learning. Paper presented at: 2022 IEEE 6th Information Technology and Mechatronics Engineering Conference (ITOEC); 2022 March 4–6; Chongqing, China.

[85] Phueakthong P, Varagul J, Pinrath N. Deep reinforcement learning based mobile robot navigation in unknown environment with continuous action space. Paper presented at: 2022 5th International Conference on Intelligent Autonomous Systems (ICoIAS); 2022 September 23–25; Dalian, China.

[86] Nossier E, Ibrahim F, Abdelwahab M, AbdelAziz M. Path planning algorithm for dynamic obstacles based on support vector machines towards autonomous navigation. Paper presented at: 2021 16th International Conference on Computer Engineering and Systems (ICCES); 2021 December 15–16; Cairo, Egypt.

[87] Yang S, Tan J, Lei T, Linares-Barranco B. Smart traffic navigation system for fault-tolerant edge computing of internet of vehicle in intelligent transportation gateway. IEEE Trans Intell Transp Syst. 2023;24(11):13011–13022.

[88] Xing D, Li J, Zhang T, Xu B. A brain-inspired approach for collision-free movement planning in the small operational space. IEEE Trans Neur Netw Learn Syst. 2022;33(5):2094–2105. 10.1109/TNNLS.2021.3111051 34520379

[89] Menon A, Natarajan A, Olascoaga LIG, Kim Y, Benedict B, Rabaey JM. On the role of hyperdimensional computing for behavioral prioritization in reactive robot navigation tasks. Paper presented at: 2022 International Conference on Robotics and Automation (ICRA); 2022 May 23–27; Philadelphia, PA, USA.

[90] Li M, Wei R, Zhang Z, Zhang P, Xu G, Liao W. CVT-based asynchronous BCI for brain-controlled robot navigation. Cyborg Bionic Syst. 2023;4:0024. 37223547 10.34133/cbsystems.0024 PMC10202181

